# Characterization of fusion genes and the significantly expressed fusion isoforms in breast cancer by hybrid sequencing

**DOI:** 10.1093/nar/gkv562

**Published:** 2015-10-10

**Authors:** Jason L. Weirather, Pegah Tootoonchi Afshar, Tyson A. Clark, Elizabeth Tseng, Linda S. Powers, Jason G. Underwood, Joseph Zabner, Jonas Korlach, Wing Hung Wong, Kin Fai Au

**Affiliations:** 1 Department of Internal Medicine, University of Iowa, 200 Hawkins Dr, Iowa City, IA 52242, USA; 2 Department of Electrical Engineering, School of Engineering, Stanford University, Stanford, CA 94305, USA; 3 Pacific Biosciences, 1380 Willow Road, Menlo Park, CA 94025, USA; 4 Department of Genome Sciences, University of Washington, 3720 15th Ave NE, Seattle WA 98195–5065, USA; 5 Department of Statistics and Department of Health Research & Policy, 390 Serra Mall, Stanford University, Stanford, CA 94305, USA

## Abstract

We developed an innovative hybrid sequencing approach, IDP-fusion, to detect fusion genes, determine fusion sites and identify and quantify fusion isoforms. IDP-fusion is the first method to study gene fusion events by integrating Third Generation Sequencing long reads and Second Generation Sequencing short reads. We applied IDP-fusion to PacBio data and Illumina data from the MCF-7 breast cancer cells. Compared with the existing tools, IDP-fusion detects fusion genes at higher precision and a very low false positive rate. The results show that IDP-fusion will be useful for unraveling the complexity of multiple fusion splices and fusion isoforms within tumorigenesis-relevant fusion genes.

## INTRODUCTION

A fusion gene is an aberrant gene formed by the concatenation of two separate genes. Gene fusion events are caused by genome translocation, interstitial deletion or chromosomal inversion. In the past three decades, important roles for gene fusion events in tumorigenesis have been established (1,2). For example, an oncogene can be upregulated by fusion with a gene containing a strong promoter (3). Alternatively, a fusion gene can be translated to a fusion protein that contains chimeric domains from two genes. Such a chimeric domain combination is likely to result in aberrant fusion protein activity or loss of function of endogenous proteins. The classic example of a fusion gene with oncogenic activity is BCR–ABL1 in leukemia (4). Because of the uniqueness of these fusion genes, they could serve as tumor-specific drug targets. However, specific inhibitors have only been developed against a few fusion genes, such as imatinib (Gleevec), which targets the BCR–ABL1 fusion gene. Moreover, there are likely many more fusion genes that remain to be detected and characterized (5). The lack of a reliable and comprehensive fusion gene detection approach is a main obstacle in fusion gene research.

The ultimate goal of fusion gene identification is to determine the transcript products of fusion genes (i.e. isoforms of fusion genes). The accurate characterization of fusion transcript sequences is the foundation of all subsequent downstream research. To accomplish this goal, an analytic tool is needed to (i) find fusion genes (i.e. the pair of involved genes); (ii) determine the fusion sites between the paired genes; and (iii) identify the expressed isoforms. With the emergence of Second Generation Sequencing (SGS), a few genome-wide fusion gene detection tools have been developed to accomplish the first two steps based on alignments of SGS short reads (6–16). However, the results of finding fusion genes and fusion sites by existing tools are still problematic. In general, a fusion gene is detected from reads that span two distinct genes, so the existing computational methods ‘split’ SGS short reads into paired fragments and find their alignments on the genome. Similarly, ‘paired-end’ SGS reads can detect gene fusions if two mates are mapped to two genes. Since gene fusion events can be interchromosomal, the search space for a split short read alignment or a paired-end read alignment is the whole genome. Considering the short read length of SGS data and the size of the human genome, the rate of ambiguous or incorrect alignment is not negligible for the following reasons: (i) misalignment due to genome variants and sequencing errors can cause incorrect detections; and (ii) multiple alignments of single reads causes uncertainty of the detections. Around 50% of the human genome (hg19) regions are repetitive, so short read alignments at these regions could be uncertain (17). Overall, the short read lengths cannot guarantee accurate alignments of split reads and paired-end reads. In addition, isoform analysis of fusion genes is still a fundamental issue which has not been explored in detail.

Third Generation Sequencing (TGS) technologies such as SMRT (Single Molecule Real Time) sequencing technology from Pacific Biosciences (PacBio) offer unique advantages for the identification of fusion genes. TGS distinguishes itself from SGS through the much longer sequencing length (>40,000 bp with average length around 10,000–15,000 bp) (18,19). Thus, these long reads can be aligned uniquely, which can ensure the reliability of finding the fusion genes. However, it remains challenging to determine the precise fusion sites of fusion genes by TGS-only data because of the high sequencing error rate (∼15%) of raw PacBio data. Although a small subset of PacBio data, termed Circular Consensus Sequence (CCS) reads, can be generated with a low error rate, their read length is limited (up to 3000 bp). The majority of PacBio reads (referred to as subreads) are too long to generate CCS reads, but they are very informative. In order to make use of PacBio data without sacrificing throughput and read length, several recent studies applied a strategy termed ‘hybrid sequencing’ to integrate PacBio long reads with high-quality SGS short reads (e.g. Illumina data). For example, the error correction tools LSC and PacBioToCA were developed to reduce errors of PacBio data by comparing with SGS short reads (20,21). In addition, we also developed a hybrid sequencing tool, IDP (Isoform Detection and Prediction) to identify and quantify gene isoforms (22). The development of IDP laid the groundwork to identify and quantify the expressed isoforms from fusion genes from hybrid sequencing data.

Herein we present IDP-fusion (http://www.healthcare.uiowa.edu/labs/au/IDP-fusion/) as a method to accurately characterize fusion genes from hybrid sequencing transcriptome data, including determination of fusion genes and fusion sites at single-nucleotide resolution and identification and quantification of expressed fusion isoforms. We applied IDP-fusion to the breast cancer cell line MCF-7 as the proof-of-concept application. MCF-7 cells are a well-established model for the discovery of fusion genes since these cells harbor more known gene fusions than any other cancer cell line (2,23). From MCF-7 hybrid sequencing transcriptome data, we identified 35 fusion genes with 56 fusion sites that were supported by at least two supporting long reads and at least two supporting short reads. As compared to the seven existing tools, TopHat-Fusion, SOAPfuse, TRUP, FusionMap, deFuse, BreakFusion and Iso-Seq, IDP-fusion has superior accuracy and a comparable sensitivity. We also applied IDP-fusion to normal human adult tissues (considered as negative controls), which revealed a very low false positive rate (FPR) for IDP-fusion. Beyond fusion detection, IDP-fusion also performs *de novo* annotation of exon-intron structure of fusion genes, including novel genes that are involved in gene fusion events. Using an RPKM threshold of 10, IDP-fusion also identified and quantitated the abundance of 30 significantly expressed fusion isoforms from 14 fusion genes. In addition to the performance analysis, we also report the identification of several novel fusion genes in MCF-7 cells, with potential implications in cancer development and response to therapy. Therefore, IDP-fusion is an innovative method that provides isoform-specific resolution of fusion genes with a low FPR, with broad applicability to fusion gene research.

## MATERIALS AND METHODS

**Figure 1.**
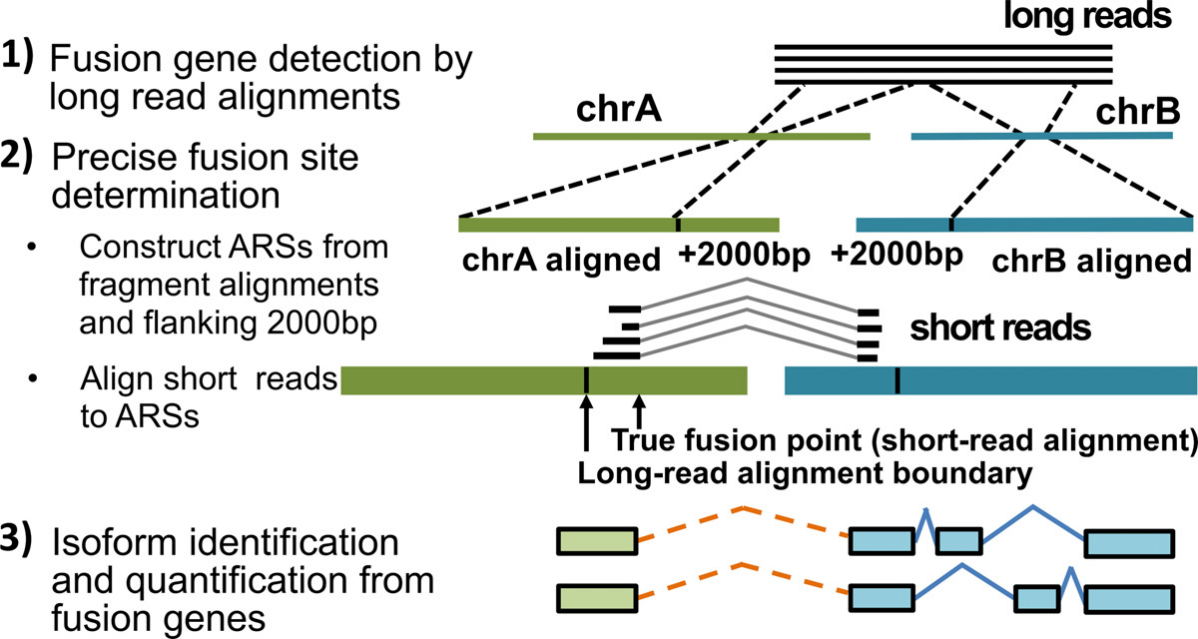
Flowchart of IDP-fusion. IDP-fusion contains three main steps: (**1**) detect fusion genes by genome-wide alignments of long reads (e.g. using GMAP); (**2**) determine precise fusion sites by alignment of short reads to Artificial Reference Sequences (ARSs) (e.g. using STAR); (**3**) identify and quantify significantly expressed isoforms, including fusion isoforms, from fusion genes.

Gene fusion events can be identified at three levels: fusion gene, fusion sites and fusion isoforms. ‘Fusion gene’ is defined as a pair of genes/gene loci that are fused. ‘Fusion sites’ refer to the connection points of the paired genes/gene loci. ‘Fusion isoform’ is an isoform of a fusion gene spanning fusion sites. Therefore, IDP-fusion includes three main steps (Figure 1 and Supplementary Figure S1): (i) detect fusion genes by aligning long reads to a reference genome; (ii) determine precise fusion sites at single-nucleotide resolution by aligning short reads to the flanking regions of long read alignments; (iii) identify and quantify significantly expressed isoforms from fusion genes by IDP modified to a fusion gene model (22). Although we applied IDP-fusion to PacBio data as the proof-of-principle application, IDP-fusion is generalizable for TGS long read data. Thus, we describe the method below for general TGS long reads instead of PacBio long reads.

### Fusion gene detection by genome-wide long read alignments

We denote long reads spanning the fusion site as ‘fusion long reads’. A fusion long read can be split into two fragments that are mappable into two gene loci. To find the paired fragment alignment of a fusion long read, we align long reads to reference genome (human genome hg19 for MCF-7 data) by GMAP (24). GMAP outputs multiple alignments of a long read, including fragment alignments. For each long read, we examine all mutant pairs of fragment alignments and define a pair of fragment alignments as a fusion gene candidate if the following conditions are met: (i) both aligned fragments are of sufficient length (>100 bp by default); (ii) two fragments are mapped to different chromosomes or on the same chromosome with a minimum distance threshold (100 kb by default) or at two different annotated genes; (iii) there is no significant overlap (100 bp by default) between two aligned fragments; and (iv) there is no significant unaligned region of fusion long reads (100 bp by default) between two aligned fragments (Supplementary Figure S2).

Considering the existence of long repetitive genomic elements and sequencing errors of TGS long read data, we applied filters to remove ambiguous fragment alignments. Each fragment that is detected as part of a fusion gene candidate is re-aligned back to reference genome separately. We define alignment identity as the number of matches over target length. If the alignment identity difference of the first and second best alignments of a fragment is small (<0.2 by default), then the corresponding fusion gene candidate is filtered out. Furthermore, we require the transcription strands of the paired fragments to be consistent. The strand of each fragment is determined by the splice detections from the fragment or the strand of the overlapping annotated gene. If the strands of the paired fragments are not consistent with their order within the long read, the corresponding fusion gene candidate is filtered out.

Next, the remaining fusion genes are annotated by reference annotation libraries, such as RefSeq. If the splice detections from the fragment alignment are annotated by a known gene library, or if the fragment alignment overlaps with a reference gene, then the fragment is assigned the gene name of the annotated splices. Otherwise, the fragment of the fusion gene is annotated by the genomic locus of the alignment.

### Precise fusion site determination by short read alignments

The ending positions of the paired alignments are not likely the true fusion sites between two genes because of the relatively high error rate of long reads. However, the true fusion sites are located in neighboring regions. The alignment of high-quality short reads to these neighboring regions allows us to find the precise fusion site(s). In order to align short reads to these neighboring regions using splice read aligners, we construct ‘Artificial Reference Sequences’ (ARSs). The genomic region of each fragment alignment is extended 2000 bp beyond the alignment ends and then two genomic regions are concatenated to be an ARS. Because of the depth of sequencing data, multiple ARSs can be generated from the same fusion locus. In order to avoid aligning short reads to these redundant ARSs, we merge the ARSs from the same fusion locus by concatenating the span ranges of the ARSs at each side (Supplementary Figure S3).

Therefore, the true fusion site is separated by a ∼4000 bp sequence gap resembling an artificial intron within ARSs. Subsequently, fusion short reads that span true fusion sites are mapped on ARSs by splice read aligners (e.g. RNA-Seq aligners). By default, we apply splice read aligner STAR to complete this step (25) which is not limited by the requirement of canonical splicing signals, because to the best of our knowledge, no solid evidence has been reported to support the usage of canonical splicing signals in fusion splices. Many studies have shown that the accuracy of the splice read aligners are of single-nucleotide resolution (25–27), so the alignments of short reads on the ARSs can determine precise fusion sites.

Finally, we also require sufficient experimental data for fusion site determination: minimum numbers of supporting long reads and minimum numbers of supporting short reads. In addition to the concern of reliability, very few supporting reads also implies the negligible expression of the corresponding fusion product. By default, we filter out the fusion sites that are supported by either only one long read or only one short read.

### Fusion isoform identification from fusion genes

To identify the fusion gene isoforms and estimate their abundance, we perform three steps: (i) call splice linkages from long read alignments; (ii) construct isoform candidates using splice linkages as seeds; (iii) estimate abundance of isoform candidates and select the ones based on the isoform abundance.

#### Non-redundant fusion splice linkages from long read alignments

First, we define splice linkages from long read alignments and then remove the redundancy of splice linkages. Long read alignments to the reference genome contain gaps, which could be genetics variants (deletions) or splices. Most deletions are short, while >99% of the splices from human genes are longer than 68 bp in RefSeq. Thus, we consider alignment gaps longer than 68 bp to be splices (Supplementary Figure S4A). The splices are further filtered out if they are not detected by short reads or annotated in existing libraries, such as RefSeq. Multiple splices detected from a single long read are defined as a splice linkage. A splice linkage within a fusion long read is called a fusion splice linkage. Fusion splices connect the regular splices of the paired fragments and are also included in splice linkages. A fusion splice is treated as a regular splice in the steps below.

Many splice linkages generated from long reads are redundant. In order to produce a set of non-redundant splice linkages, we define two types of exon boundaries within each gene locus: (i) all splice sites of novel splices and annotated splices; (ii) 5′ and 3′ ends of all annotated gene isoforms. Two flanking bases around exon boundary *P_i_* are labeled as $P_{i}^ -$ and $P_{i}^ +$ (Supplementary Figure S4B). Then, splice linkage can be denoted by a set of exon boundaries that are covered by a long read. If the splice linkage of a long read is identical or is a subset of the other, then it is considered redundant and removed.

#### Fusion isoform candidate construction

**Figure 2.**
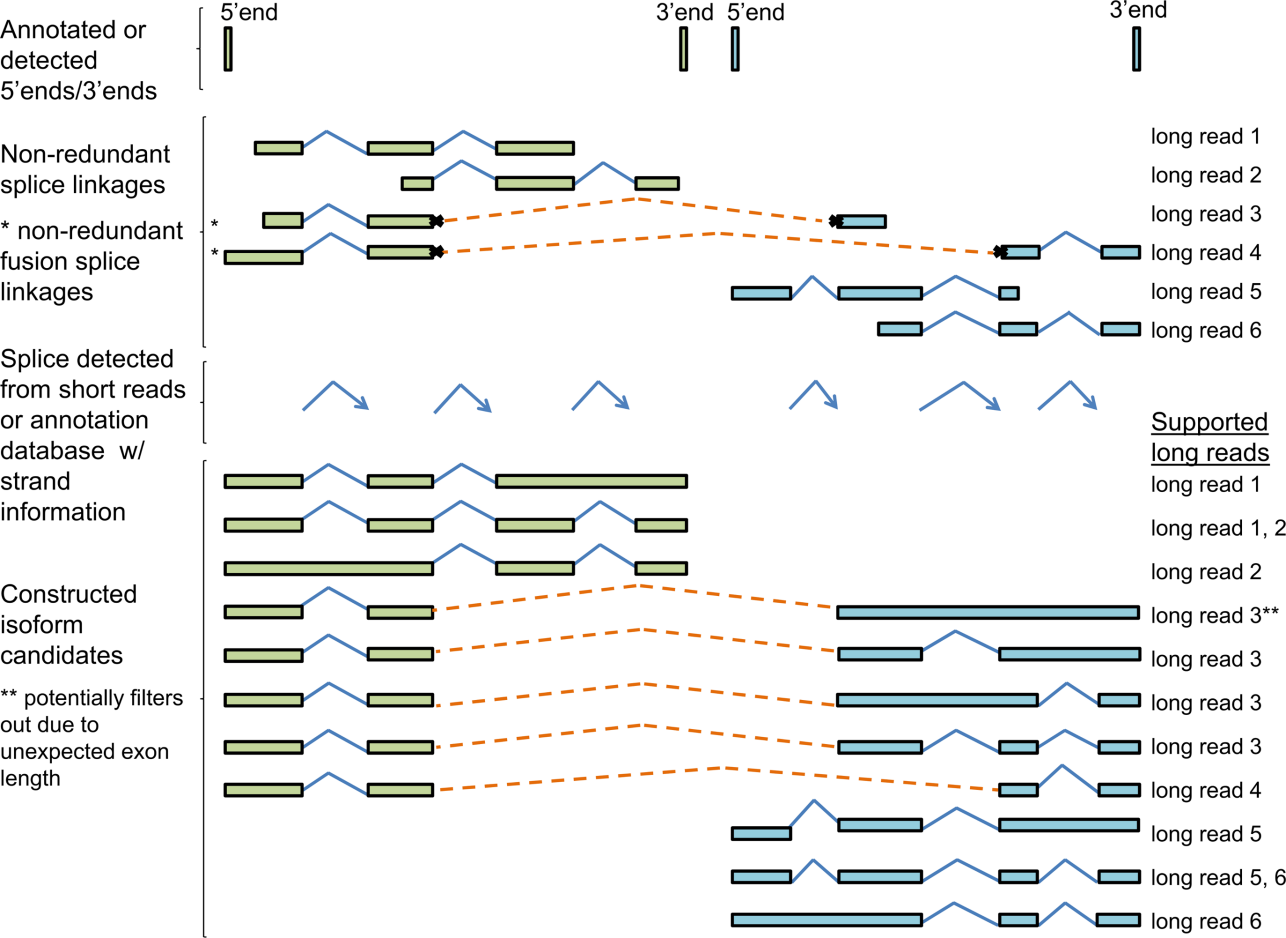
Isoform candidate constructions from fusion genes. Isoform candidate constructions contain three structural units: 5′/3′ ends, non-redundant splice linkages (including fusion splice linkages) and splices. All possible splice combinations are included until reaching the 5′ end and 3′ end. Both regular isoforms and fusion isoforms are constructed.

We construct isoform candidates by combining three structural units: non-redundant splice linkages, splices and 5′/3′ ends. Using the non-redundant splice linkage as the seed, upstream and downstream splices with the consistent strand are added to fill the gap between the possible 3′ and 5′ ends (Figure 2). For a fusion gene, we construct both regular isoform candidates within original genes and fusion isoform candidates across original genes. To construct fusion isoform candidates, the 5′ end of the 5′ gene and 3′ end of the 3′ gene are used.

The non-redundant splice linkages are generated as above. The splice pool includes annotated splices and novel splices detected from short reads. The pool of 5′/3′ ends is built from PacBio data or annotation libraries. The PacBio data were collected from polyA-selected mRNA libraries, so long read alignments can detect 3′ ends by finding polyT/A sequences at the ends of reads. Optionally, 5′ ends detected from CAGE data can be also used (28,29). In addition, annotated 5′/3′ ends from RefSeq, Ensembl, KnownGene and Gencode are included (30–33).

#### Abundance estimation of isoform candidates and selection of significantly expressed fusion isoforms

To estimate the abundance of isoform candidates, we apply the Poisson model of short read coverage and the MLE solution developed by Jiang and Wong (34).

Next, we perform receiver operator characteristic (ROC) analysis to find a threshold of isoform abundance to select isoform candidates under a given control of FPR. Alternatively, we can manually set a threshold of abundance to select significantly expressed isoforms and the corresponding FPR can be computed. Isoform abundance is the classifier parameter in ROC analysis to calculate sensitivities versus FPR. First, we perform ROC analysis on only the non-fusion genes to find a threshold of abundance under a given FPR. Next, we select the fusion isoform candidates with this threshold from fusion genes.

The sets of true positives and true negatives in ROC analysis are constructed as follows. We regard isoform candidates as true positives if they are directly detected by full splice linkages. A library of Two-Consecutive-Splice-Linkages (TCSL) is generated from RefSeq, Ensembl, Gencode, KnownGene and EST (30–33,35) (Supplementary Figure S5). These reference libraries are generated based on hundreds of thousands of data from different samples, different techniques and different research groups. Although some data may only cover fragments of transcripts, most possible TCSLs in nature are likely to be seen in these libraries. Thus, we regard an isoform candidate as a true negative if any TCSL of this isoform is not supported by the TCSL library.

### Alignment of short reads to ARSs

The STAR aligner (v2.3.0e) was used to map short reads to ARSs. Alignment parameters were set to report all possible alignments of the reads, including multiply mappable hits, with a minimum overhang of 12 bp for any type of splice junction. The parametric settings are as follows: [outSJfilterCountUniqueMin = 0, outSJfilterCountTotalMin = 1, outSJfilterOverhangMin = 12, alignTranscriptsPerReadNMax = 63200 (the number of ARSs), outFilterMultimapNmax = 63200, alignWindowsPerReadNmax = 63200, alignIntronMin = 68, alignIntronMax = 400000].

## RESULTS

The default PacBio transcriptome data process pipeline, Iso-Seq, is not used in IDP-fusion. Based on the raw data, we obtained 1,984,211 CCS reads and 5,441,586 subreads (see Supplementary Materials and Methods, accession ID: PRJNA277461) of human MCF-7 transcriptome from PacBio *RS*II platforms. We downloaded 183,946,388 101 bp Illumina short reads from GSE49831 (36). By inputting both PacBio long reads and Illumina short reads to the error correction tool LSC (20), 7,425,797 error-corrected PacBio long reads were output, 6,855,328 of which were mapped to the human genome (hg19). The corrected long reads and short reads were input into IDP-fusion for detection of fusion genes, fusion sites and fusion isoforms, which are discussed in order below. By requiring ≥2 supporting long reads and ≥2 supporting short reads, IDP-fusion identified 56 distinct fusion sites from 35 fusion genes. For these fusion genes, 100 significantly expressed (RPKM > 10) isoforms were predicted with FPR < 3.8%, 30 of which were fusion isoforms spanning fusion sites.

### Fusion gene detection by IDP-fusion and comparison with SGS-only and TGS-only tools

**Figure 3.**
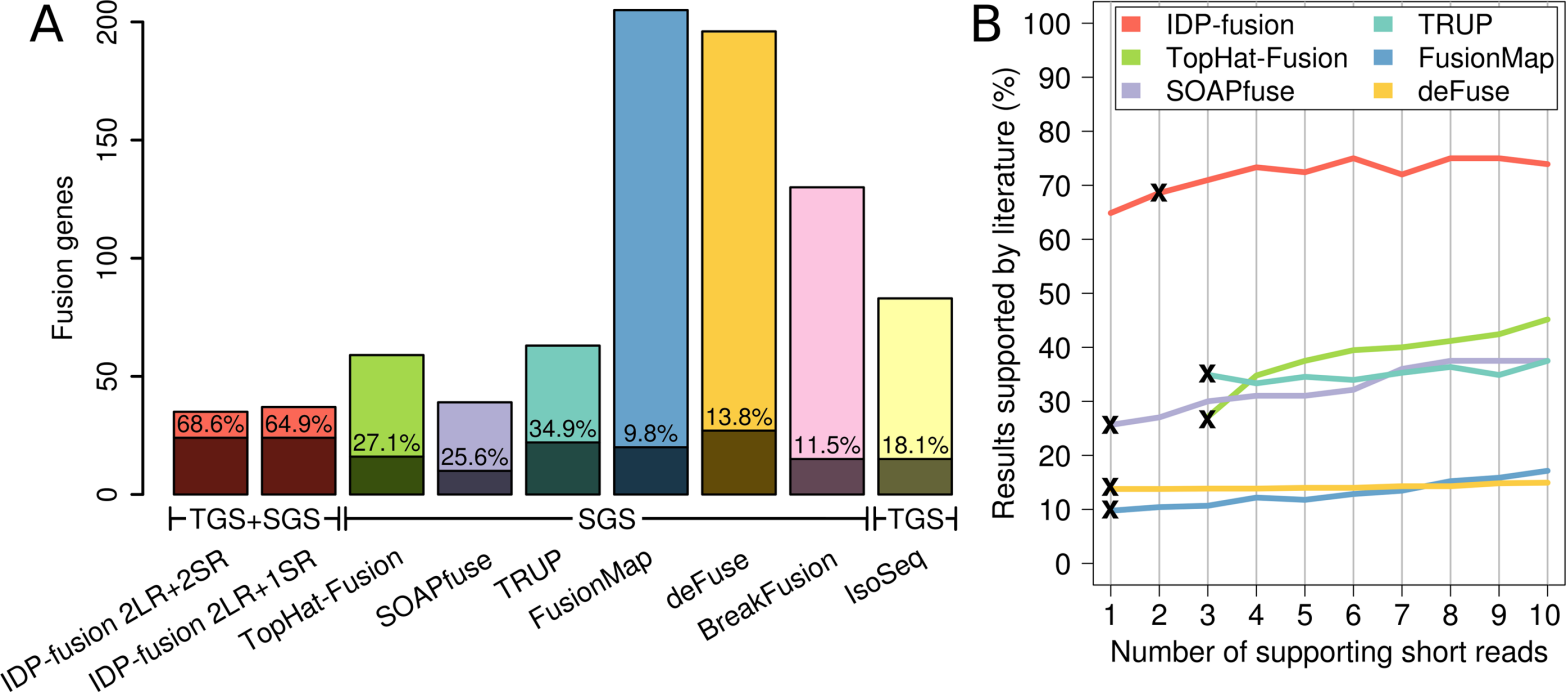
Precisions of fusion gene detections by IDP-fusion, six SGS-only methods and a TGS-only method. (**A**) The total numbers of fusion genes detected from MCF-7 cells by IDP-fusion, six SGS-only methods and Iso-Seq (TGS-only method) and the corresponding numbers of the gold standard fusion genes are shown by stacked bars. The precisions are also shown as the rates of the gold standard fusion genes detected. Two modes of IDP-fusion, using either ≥2 supporting long reads (LR) and ≥1 supporting short read (SR) or ≥2 supporting long reads and ≥2 supporting short reads (default settings) are compared with six SGS-only methods that were run under their default settings and Iso-Seq method requiring ≥5 full-length long reads. (**B**) The precision of fusion gene detection by IDP-fusion is higher than SGS-only methods, regardless of increase of the minimum numbers of supporting short reads. The precision of the default setting for each method is labeled by ‘x’. Note that BreakFusion is not shown because the software does not output the number of supporting reads for each fusion site.

With the same MCF-7 data, we ran the SGS-only methods TopHat-Fusion, SOAPfuse, TRUP, FusionMap, deFuse and BreakFusion (7–9,12,15–16), which predicted 59, 39, 63, 205, 196 and 130 fusion genes respectively. To further compare the differences in sensitivity and precision of these tools, we defined a gold standard set of fusion genes in MCF-7 cells that were independent of the development of computational methods. Specifically, we collected a set of 71 fusion genes (Supplementary Table S1) based on 7 non-computational publications which were validated by either PCR and/or Sanger sequencing (37–43). From 35 fusion genes detected by IDP-fusion, 68.57% (24/35) were supported by the gold standard. In contrast, only 27.12% (16/59), 25.64% (10/39), 34.92 (22/63), 9.76% (20/205), 13.78% (27/196) and 11.54% (15/130) of the output from TopHat-Fusion, SOAPfuse, TRUP, FusionMap deFuse and BreakFusion were supported by the gold standard (Figure 3A and Table 1). When relaxing the requirement of supporting short reads from ≥2 to ≥1, IDP-fusion identified 37 fusion genes, with 24 in the gold standard set. The precision of 64.86% was still considerably higher than SGS-only tools. While IDP-fusion shows superior precision, the sensitivity is still comparable with SGS-only tools. In total, both modes of IDP-fusion detected 24 gold standard fusion genes, while the six other tools found 16, 10, 22, 20, 27 and 15, respectively. These results demonstrate that IDP-fusion can achieve a comparable sensitivity to SGS-only methods with higher precision gained from incorporating long reads in the search.

**Table 1.** Performance comparison of fusion gene detection methods

Type	Method	Gold standard fusion genes detected	Sensitivity^b^	Total fusion genes detected	Precision	F-score
SGS + TGS	IDP-fusion 2LR + 2SR^a^	24	33.80%	35	68.57%	0.4528
	IDP-fusion 2LR + 1SR	24	33.80%	37	64.86%	0.4444
SGS	TopHat-Fusion	16	22.54%	59	27.12%	0.2462
	SOAPfuse	10	14.08%	39	25.64%	0.1818
	TRUP	22	30.99%	63	34.92%	0.3284
	FusionMap	20	28.17%	205	9.76%	0.1449
	deFuse	27	38.03%	196	13.78%	0.2022
	BreakFusion	15	21.13%	130	11.54%	0.1493
TGS	IsoSeq	15	21.13%	83	18.07%	0.1948

^a^LR: long read; SR: short read.

^b^Sensitivity is defined as the detection rate of the whole gold standard set containing 71 fusion genes.

The uniqueness of a fusion long read alignment depends on the length of the shorter flanking fragment (referred to as Short Fragment Length, SFL). For a fusion site and thus the corresponding fusion gene, the maximum SFL (referred to as MSFL) among all supporting long reads is an indicator of the information that can be obtained from long reads. Among fusion sites detected by IDP-fusion in the MCF-7 dataset, the median MSFL was 320.5 bp (Supplementary Table S2). In contrast, the theoretical largest MSFL for 200 bp SGS data is only 100 bp, because SFL for SGS data cannot be longer than half of a read length. Therefore, it is more reliable to pinpoint fusion genes by long read alignments. In addition, the information provided by long read alignments cannot be attained by increasing short read depth. When we increased the requirement of supporting short reads, the gold standard rates of IDP-fusion, TopHat-Fusion, SOAPfuse and FusionMap increased, however, the significant gap between IDP-fusion and SGS-only methods remained. Even when requiring ≥10 supporting short reads, the precisions of SGS-only methods were still subpar to IDP-fusion requiring ≥1 short reads (Figure 3B).

Besides the SGS-only methods, we compare IDP-fusion to the TGS-only analysis pipeline Iso-Seq. Among the fusion genes detected by Iso-Seq, 18.07% (15/83) were found in the gold standard set (Figure 3A and Table 1), which is also lower than IDP-fusion. In addition, IDP-fusion can identify and report fusion genes based on TGS-only data without utilizing short reads (Supplementary Table S3). The results of the TGS-only mode of IDP-fusion are comprised of 6.18% (26/421) gold standard fusion genes. The result above indicates that the requirement of short read support in our IDP-fusion method has only a minor effect on sensitivity while greatly improving precision, as the sensitivity changes from 36.62 to 33.80% and the precision goes up from 6.18 to 68.57% (Supplementary Table S4).

### Determination of fusion sites by IDP-fusion

**Figure 4.**
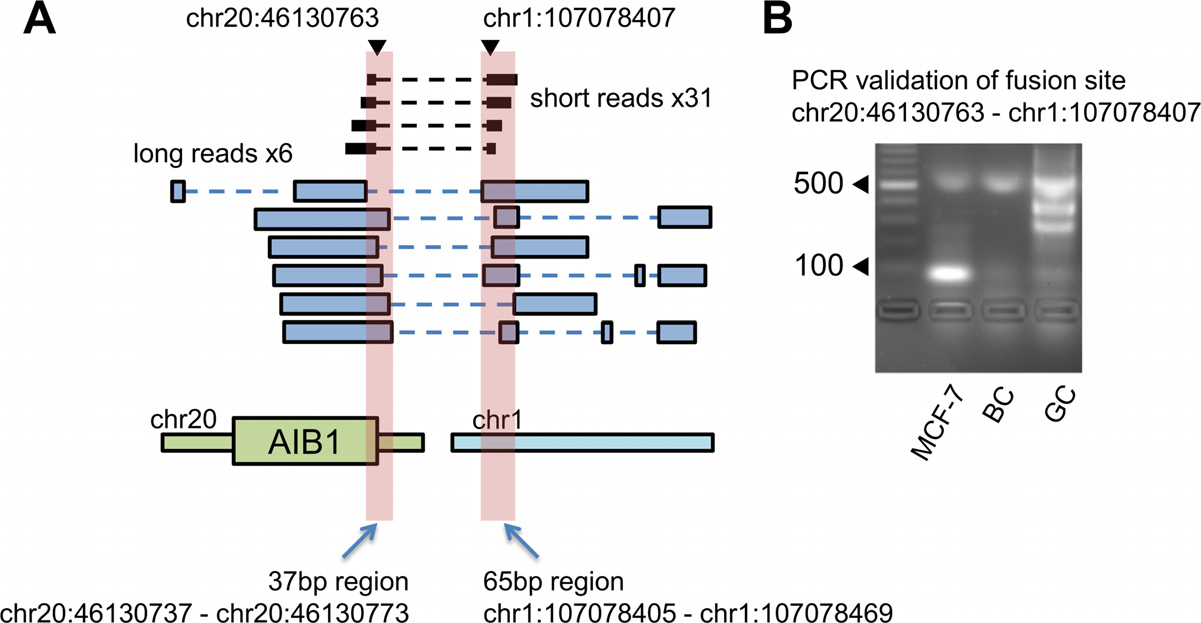
Precise fusion site determination of the fusion gene AIB1–chr1:107078407. (**A**) A fusion between AIB1 and an unannotated region of chromosome 1 was detected by six long reads (blue blocks), but the long read alignment ends are not in agreement on the precise fusion site. The alignment ends of the long read fragments span 37 and 65 bp at two sides, respectively. IDP-fusion aligned 31 short reads (black blocks) to the fusion site precisely at chr20:46130763–chr1:107078407. In particular, chr20:46130763 is the 3′ end of the first exon of AIB1 and the canonical splicing signal is found in this fusion site. (**B**) The fusion site chr20:46130763–chr1:107078407 was PCR validated from MCF-7 cDNA, but not healthy breast cDNA (BC) or a genomic control (GC).

Although the uniqueness of long read alignments improved fusion gene detection, the high error rate of the TGS long reads prevents the precise determination of fusion sties. For example, for the fusion site chr20:46130763–chr1:107078407 in the fusion gene AIB1–chr1:107078407, the ending positions of six PacBio long read alignments varied in a 37 bp region (chr20:46130737–chr20:46130773) and a 65 bp region (chr1:107078405–chr1:107078469), rather than all aligning exactly at chr20:46130763 and chr1:107078407, respectively. IDP-fusion aligned 31 short reads at this fusion site between chr20 and chr1 (Figure 4A) precisely at chr20:46130763 and chr1:107078407, which were further validated by PCR and Sanger sequencing (Figure 4B). In total, six fusion sites were detected in the fusion gene AIB1–chr1:107078407, four of which have been validated previously in the literature (37). In contrast, none of these validated fusion sites were reported by SGS-only tools.

As illustrated in the fusion gene AIB1–chr1:107078407, TGS long reads can pinpoint the fusion sites in a small range rather than single-nucleotide resolution, due to their relatively higher error rate and lower coverage. However, SGS-only data is also not able to capture some fusion sites, especially when the fusion sites are in repetitive regions. For example, for the fusion gene ATXN7–chr1:106735582, IDP-fusion determined the fusion site at chr3:63933690 and chr1:106735582, which was also validated by Inaki *et al.*, using PCR (37). Six SGS-only tools did not detect this fusion site and the corresponding fusion genes, because the flanking regions of chr3:63933690 and chr1:106735582 are repetitive (Supplementary Table S2). However, the MSFL of supporting long reads at this fusion site is 621 bp which covers uniquely mappable regions. The artificial reference sequence (ARS, see ‘Materials and Methods’ section) library generated by long read alignments is much smaller than the whole genome, so that short reads can be more reliably mappable to find the fusion site precisely. In the fusion gene ENSG00000224738-VMP1, IDP-fusion determined an extra fusion site, chr17:57184952–chr17:57911373, in addition to two sites that were also detected by SGS-only methods. Similarly, the flanking regions of this hit are repetitive, but the MSFL of the corresponding supporting long reads is 615 bp, indicating the long read alignments are unique. Therefore, IDP-fusion provides a comprehensive determination of fusion sites, thereby allowing subsequent identification and quantification of isoforms of the fusion gene. A complete isoform library is fundamental to quantify fusion isoforms and fusion genes.

**Figure 5.**
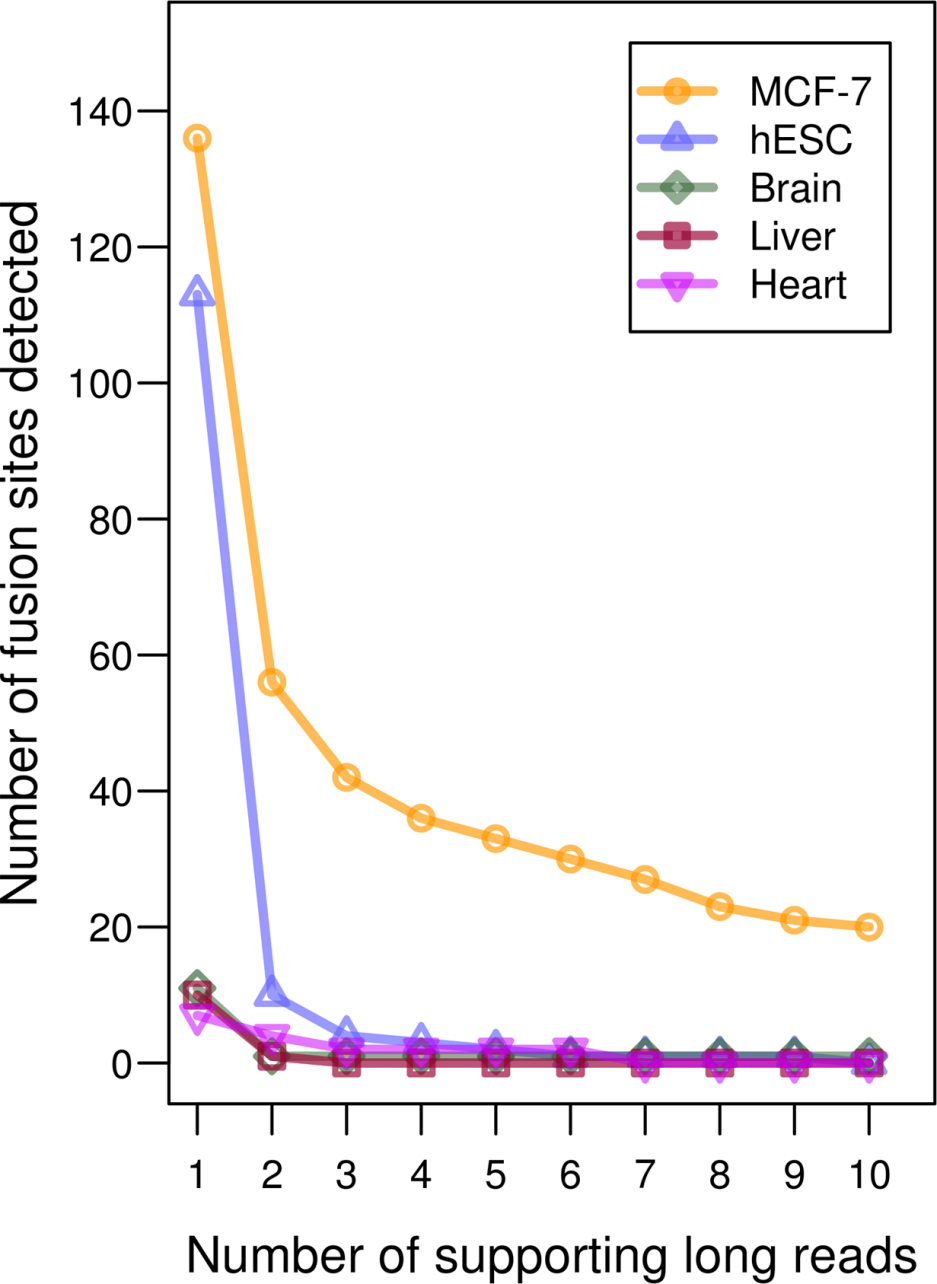
The numbers of fusion sites determined from MCF-7 cells and four normal samples. Compared with the breast cancer cells MCF-7, four negative controls (human embryonic stem cell line/hESC, and human brain, liver and heart) are expected to have negligible gene fusion events. As increasing the requirement of supporting long reads, the numbers of fusion sites decreases. The dramatic drop occurs at the requirement of ≥2 supporting long reads. The fusion sites determined from the negative controls are negligible.

It remains possible that false fusion site detection can still be called by artificial chimeras produced during the cDNA preparation process and misalignments caused by sequencing errors and repetitive elements. To determine if IDP-fusion produces such false positive hits, we tested IDP-fusion on human brain, heart and liver tissues and human embryonic stem cells (hESCs, H1 cells) as negative controls (See Supplementary Materials and Methods). The hybrid sequencing data of hESCs were downloaded from GSE51861 (22). Compared with MCF-7 cells, the fusion sites predicted from the normal tissues were negligible. By the default requirements of ≥2 supporting long reads and ≥2 supporting short reads, 1, 4 and 1 fusion sites were predicted from human brain, heart and liver samples by IDP-fusion, respectively, while 56 fusion sites were predicted in MCF-7 cells (Figure 5). Ten hits were found in hESCs. Since hESCs are subjected to many passages in culture, they may have undergone genomic rearrangements and contain some true fusion transcripts. Thus, the FPR of IDP-fusion is very low.

To identify the optimal requirements, we tuned the requirement of supporting reads. We observed a dramatic elimination of false positive hits by increasing the minimum number of supporting long reads from 1 to 2, regardless of the number of supporting short reads (Figure 5 and Supplementary Figure S6). After setting the requirement of ≥2 supporting long reads, we found a reasonable improvement by increasing the minimum number of supporting short reads to 2, with little improvement from 2 to 3 supporting short reads. F-score is a measure of accuracy that accounts for both sensitivity and precision, and is defined as the harmonic mean of the sensitivity and precision (44). The F-score of fusion gene detections for ≥2 supporting short reads was highest at 0.4528 (Supplementary Figure S7). Thus, IDP-fusion requires ≥2 supporting long reads and ≥2 supporting short reads by default.

**Figure 6.**
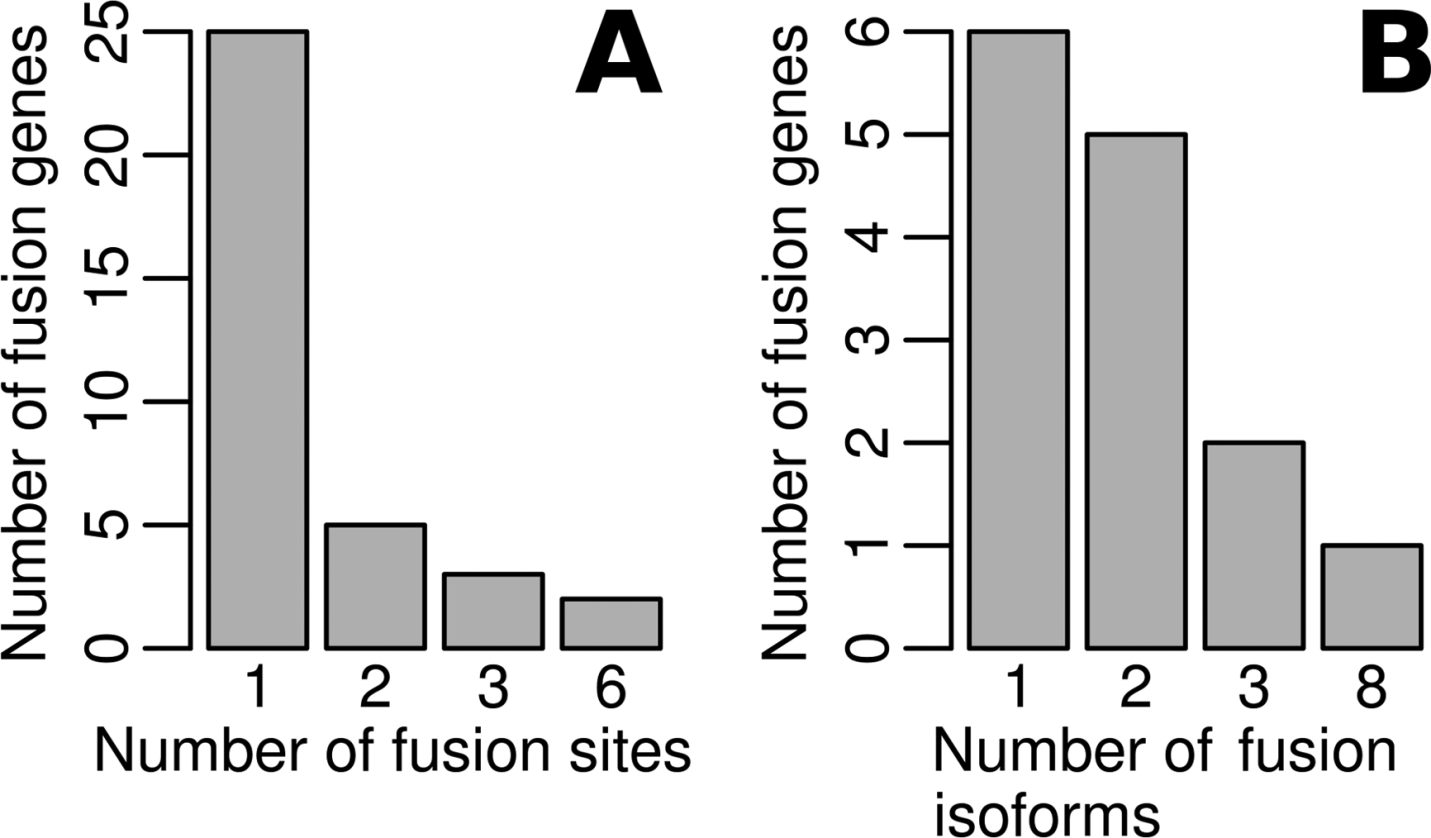
The distribution of fusion sites and fusion isoform counts in fusion genes of MCF-7 cells. (**A**) Single fusion sites were determined from 25 fusion genes and multiple fusion sites in the other 10 fusion genes. In particular, six fusion sites were determined in both fusion genes AIB1–chr1:107078407 and BCAS4–BCAS3. (**B**) Significantly expressed fusion isoforms (RPKM > 10) were identified from 14 fusion genes. Eight significantly expressed fusion isoforms were identified from BCAS4-BCAS3 (Figure 7A).

Although fusion sites may represent the chromosomal translocation points of fusion genes, multiple fusion sites within the same fusion gene are likely due to alternative splicing across two genes during transcription. Among 56 fusion sites determined by IDP-fusion, 45 (80.36%) contain canonical splicing signals (Supplementary Table S2). These data suggest that the regular alternative splicing mechanism may still occur across the paired genes involved in the fusion event. Thus, we denote such a fusion site as a ‘fusion splice’. We observed multiple fusion splices in 10 of 35 fusion genes detected by IDP-fusion (Figure 6A). In addition to AIB1–chr1:10778407, BCAS4–BCAS3 is the other fusion gene that expressed 6 fusion splices. Although BCAS4–BCAS3 has been investigated in depth, IDP-fusion is the first to discover a comprehensive set of fusion sites/fusion splices (45). These alternative fusion splices can generate multiple fusion isoforms, which render more diversity and higher complexity to the fusion products.

### Fusion isoform identification and quantification by IDP-fusion

IDP-fusion identified and quantified 100 significantly expressed isoforms (RPKM > 10) from 22 fusion genes. Of these significantly expressed isoforms, 30 represented fusion isoforms that span the fusion sites. These 30 significantly expressed fusion isoforms were derived from 14 fusion genes, whereas the other 8 fusion genes did not express high-abundance fusion isoforms (RPKM > 10). These data demonstrate that some genes undergo normal expression after the fusion event and may dominate the overall gene abundance. In this scenario, the chromosomal translocation site is likely outside of the gene locus.

**Figure 7.**
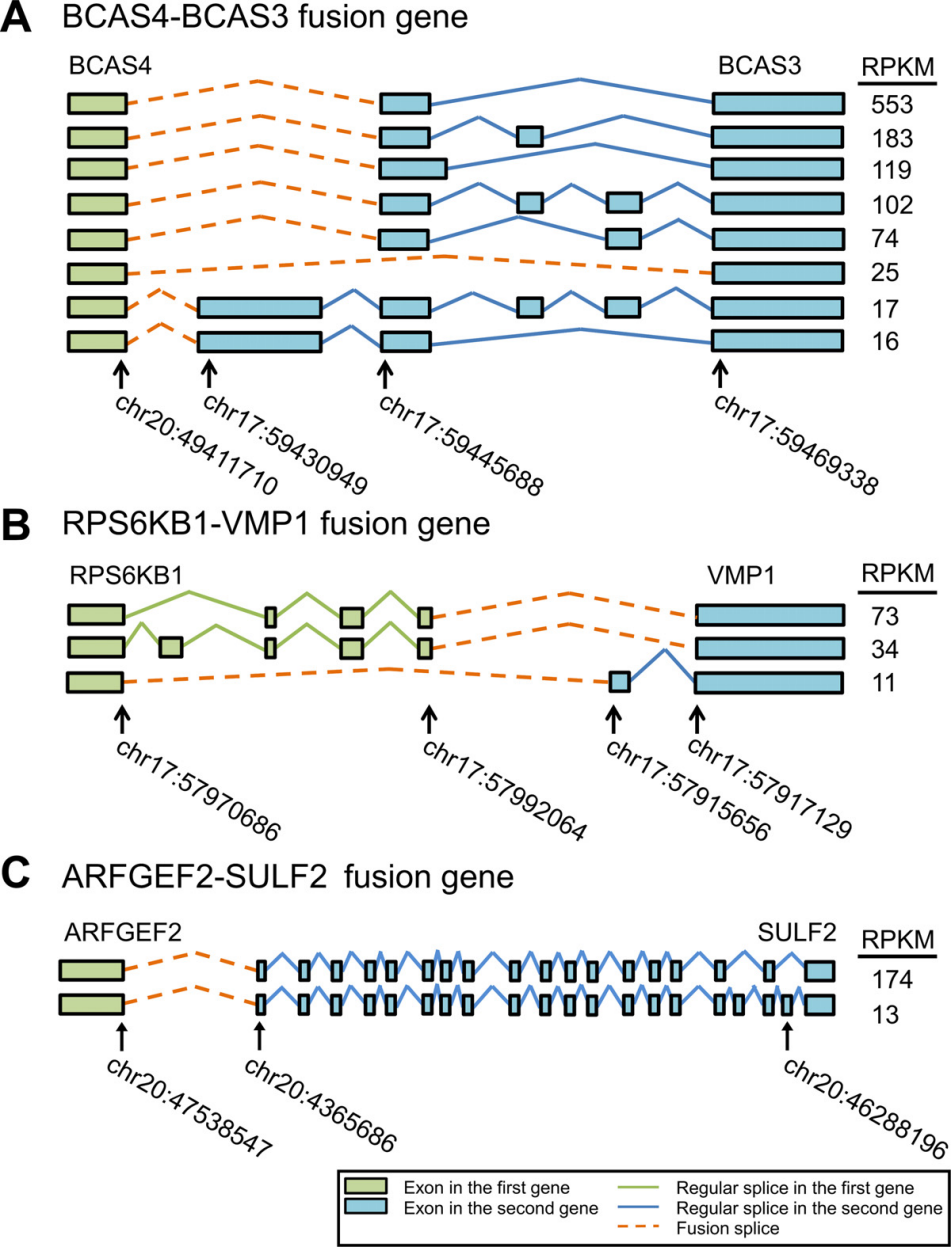
Illustration of fusion isoforms occurring through alternative fusion splices or alternative regular splices. (**A**) Eight significantly expressed fusion isoforms were identified and quantified in the fusion gene BCAS4-BCAS3, sharing three fusion splices. Besides alternative fusion splices, the regular alternative splices within BCAS3 also contribute to the fusion isoform generation. (**B**) Three significantly expressed fusion isoforms were identified and quantified in the fusion gene RPS6KB1–VMP1, sharing two fusion splices. Besides alternative fusion splices, the regular alternative splices within both RPS6KB1 and VMP1 also contribute to the fusion isoform generation. (**C**) Two significantly expressed fusion isoforms were identified and quantified in the fusion gene ARFGEF2–SULF2, sharing only one fusion splice. The diversity of fusion isoform expression is driven by the regular alternative splicing of SULF2.

Focusing on fusion isoform expression, we found that both fusion splices and regular splices can provide extensive expression from diverse fusion isoforms. Specifically, we observed that 6 fusion genes only had one significantly expressed fusion isoform, while eight expressed more than one (Figure 6B). In particular, eight significantly expressed fusion isoforms were identified in the well-known fusion gene BCAS4–BCAS3 (Figure 7A). Among eight fusion genes with multiple significantly expressed fusion isoforms, IDP-fusion detected multiple fusion splices in six fusion genes (Supplementary Table S5). For example, three fusion splices were found from eight significantly expressed fusion isoforms in BCAS4–BCAS3 (Figure 7A). Five fusion isoforms of BCAS4–BCAS3 share a fusion splice and dominate the total abundance of all significantly expressed fusion isoforms (94.6% in total). Although the other two fusion splices shared by the other three fusion isoforms only contributed 2.3 and 3.1%, respectively, to the total abundance of BCAS4–BCAS3 fusion isoforms, their expression was not negligible (RPKM > 10). Similarly, IDP-fusion found that another well-known fusion gene RPS6KB1–VMP1 expressed three fusion isoforms that were created by two distinct fusion splices with relative abundance of 90.8 and 9.2% (Figure 7B).

We also found that the diversity of fusion isoform expression could be generated by regular alternative splicing within original genes rather than through alternative fusion splices. In the fusion gene ARFGEF2–SULF2, although two fusion splices were determined, two significantly expressed fusion isoforms were generated from one fusion splice chr20:47538547–chr20:46365686 due to alternative splicing within SULF2 (Figure 7C). No fusion isoform was predicted from the fusion splice chr20:47540643–chr20:46378557, since only three short reads were aligned to it while 620 short reads supported the main fusion splice.

**Figure 8.**
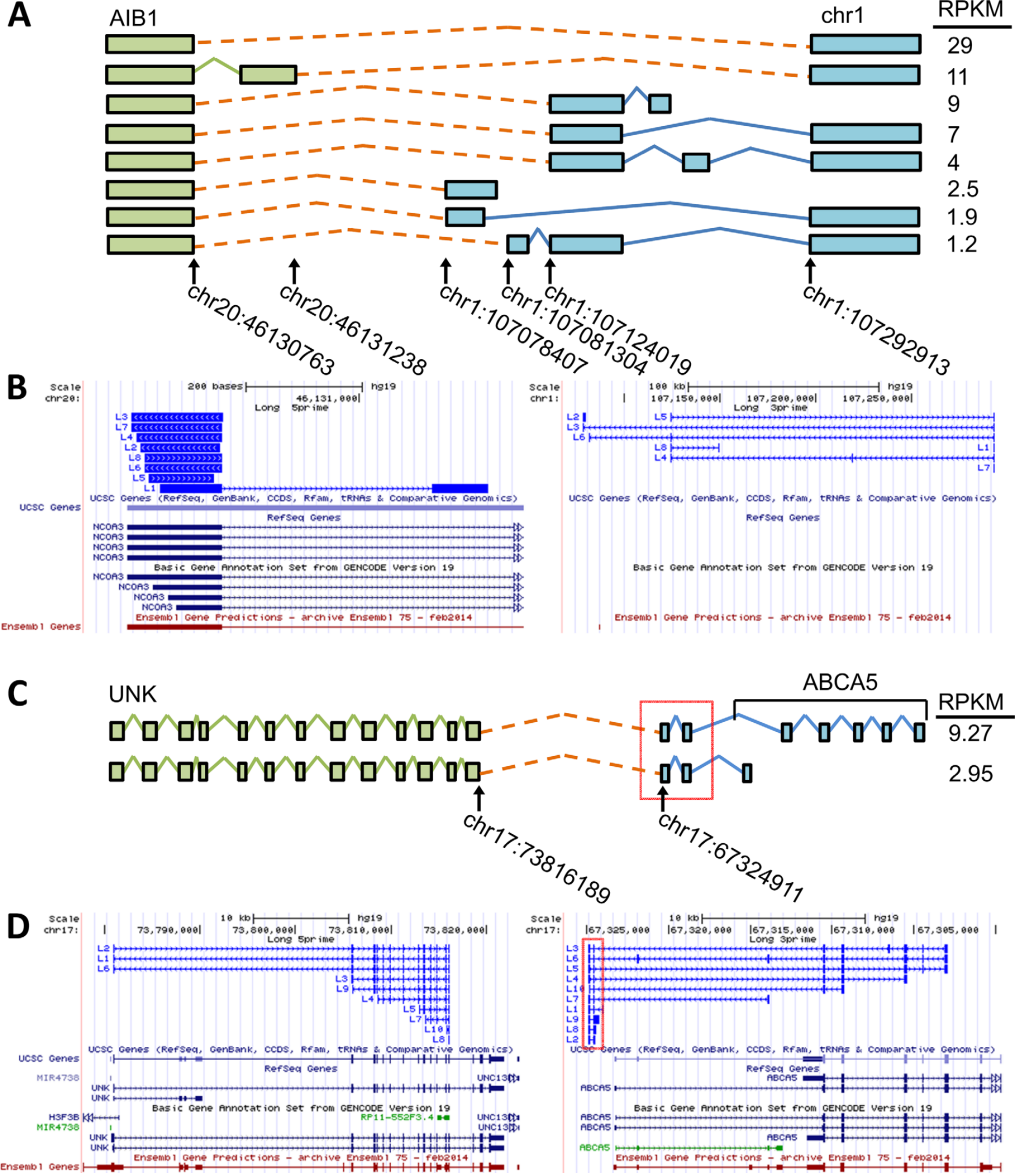
IDP-fusion detected and annotated fusion genes with novel gene and novel exons involved. (**A**) IDP-fusion detected the fusion gene between AIB1 and an unannotated region in chromosome 1. Eight fusion isoforms were estimated at the modest abundance (RPKM > 1) and contain seven novel exons (blue) annotated by IDP-fusion. (**B**) A selection of fusion long read alignments (bright blue) contributing structural information to isoform reconstruction is displayed in line with four reference annotations libraries: UCSC, RefSeq, GENCODE and Ensembl. The long read fragment alignments to AIB1 gene locus are shown at the left and the ones to the novel gene locus at the right. The long read alignments annotated a novel gene with seven exons not reported by the reference annotation libraries. (**C**) IDP-fusion detected the fusion gene UNK–ABCA5 and two corresponding fusion isoforms with RPKM > 1. This fusion gene involves two novel exons (in red box) upstream of ABCA5. (**D**) A selection of fusion long read alignments (bright blue) contributing structural information to isoform reconstruction is displayed in line with four reference annotations libraries: UCSC, RefSeq, GENCODE and Ensembl. The long read fragment alignments to UNK gene locus are shown at the left and the ones to ABCA5 gene locus at the right. The long read alignments detected two novel exons (in red box), while they are not reported by the reference annotation libraries. Note that ABCA5 is transcribed from the reverse strand. To show the fusion gene in the correct order, the browser figure of the ABCA5 gene locus is flipped. Please refer to Figure 7 for a description of figure elements.

IDP-fusion can discover fusion genes that contain novel genes, and it can identify their isoforms without depending upon a reference annotation library. This advantage is derived from the long read alignments that can determine the exon–intron structure of novel genes. For example, IDP-fusion detected a fusion event between the first 5′ exon of AIB1 and an intergenic region 1p21.1. Seven exons were determined at this novel gene by the alignments of 57 long reads (Figure 8A and B). Together with five fusion splices, IDP-fusion predicted eight fusion isoforms with at least a modest abundance (RPKM > 1), two of which passed the RPKM threshold of ten and were referred to as ‘significantly expressed’. IDP-fusion *de novo* annotated seven exons of this novel gene, which are expressed in these fusion isoforms. The same results were not detected by six SGS-only methods. IDP-fusion also found novel exons of annotated genes. For example, two fusion isoforms of the novel fusion gene UNK–ABCA5 were of modest abundance (RPKM 9.27 and 2.95). Both contain two novel exons upstream the known 5′ end of ABCA5 (Figure 8C). No regular isoforms of ABCA5 were detected that contain these novel exons and they are not reported by any publications or annotation libraries (Figure 8D). Therefore, the novel exons are likely only expressed in the fusion product UNK–ABCA5 rather than normal expression of ABCA5.

### Discovery of tumorigenesis-relevant fusion genes and fusion isoforms

IDP-fusion discovered multiple tumorigenesis-relevant fusion genes (Table 2) from MCF-7 cells (41,45–52). Regardless of whether these fusion genes have been previously reported, IDP-fusion's results led to unprecedentedly comprehensive characterization of these fusion genes, including revealing multiple fusion sites and isoforms. BCAS4–BCAS3 is a well-known and highly expressed fusion gene in breast cancer (45). BCAS4 and BCAS3 are located at 20q13 and 17q23, respectively, regions which undergo amplification, overexpression and fusion in breast cancers. Thus, expression of the fusion gene BCAS4–BCAS3 is indicative of genome rearrangement and possibly amplification of that fusion region, also giving rise to the high expression of other genes in that locus. BCAS4–BCAS3 has been detected in many breast cancer samples, yet very few studies reported multiple fusion sites and expressed isoforms. BCAS3 is a large gene containing more than 20 exons and spanning ∼715 kb, so interpretation of the complexity of the fusion sites and fusion isoforms with BCAS4 is challenging. IDP-fusion determined six fusion sites, including a novel site at a repetitive region (Supplementary Table S2) and eight fusion isoforms with significant expression (RPKM > 10) (Supplementary Table S5).

**Table 2.** The tumorigenesis-relevant fusion genes detected from MCF-7 cells by IDP-fusion

Fusion gene	5′ location	3′ location	Comments
ABCA5–PPP4R1L	17q24.3	20q13.32	ABCA5—Ovarian cancer prognosis (56)
AIB1–chr1:107073407	20q13.12	1p21.1–1p13.13	AIB1—Estrogen-dependent transcription, affect treatment of tamoxifen (46–48)
ARFGEF2–SULF2	20q13.13	20q13.12	SULF2—Cell proliferation and survival, splice-variants and pancreatic cancer prognosis (41,55)
BCAS1–chr20:46735931	20q13.2	20q13.13	BCAS1—Breast cancer prognosis (49)
BCAS4–BCAS3	20q13.13	17q23.2	Expression in breast cancer cell lines (45)
TBL1XR1–RGS17	3q26.32	6q25.2	TBXL1R1—Cell migration and invasion (50)
			RGS17—Chemoresistive ovarian cancer (51)
TPD52L2–chr17:60952559	20q13.33	17q23.2	TPD52L2—Breast cancer proliferation (52)
UNK–ABCA5	17q25.1	17q24.2	ABCA5—Ovarian cancer prognosis (56)

Another example of high complexity interpreted by IDP-fusion is AIB1–chr1:107078407. AIB1 is a known oncogene (53) and high expression of AIB1 in breast cancer is associated with a significant decrease in mortality and in recurrence following tamoxifen treatment (46,47). Four fusion sites for AIB1–chr1:107078407 have been previously reported by Inaki in a supplementary table (37) and IDP-fusion discovered another two new fusion sites in this fusion gene. More importantly, IDP-fusion determined the exon–intron structure of the novel gene involved and identified the fusion isoforms and their abundance (Figure 8A and B). These findings could form the basis of developing novel drug targets against AIB1.

Other tumorigenesis-relevant fusion genes identified by IDP-fusion include ARFGEF2–SULF2, ABCA5–PPP4R1L and UNK–ABCA5 (Table 2) (41). SULF2 is a negative regulator of cell growth and angiogenesis (54). Hampton *et al.* showed that SULF2 knockdown increases cell proliferation and survival (41). Roop *et al.* observed an increased expression of a splice-variant of SULF2 in pancreatic cancer (55), highlighting the prognostic value of evaluating tumors at the isoform level of SULF2. The fusion product we identified only included one 5′ exon of ARFGEF2, so it may either drive expression of the SULF2 fusion or perhaps more likely, this fusion serves to disrupt SULF2 function. IDP-fusion found two fusion sites for this fusion gene, one of which contributes to two significantly expressed isoforms. Low expression of ABCA5 was reported to be indicative of a poor outcome in ovarian cancer, with increased expression associated with an improved overall survival (56). IDP-fusion found the known fusion products between ABCA5 and PPP4R1L, and also discovered the completely novel fusion products between UNK and ABCA5, which contain two novel exons upstream of ABCA5 and three fusion sites. To the best of our knowledge, no existing publication has reported the fusion gene UNK–ABCA5, and it was not detected by three SGS tools. Involvement of ABCA5 in two fusion events with distinct genes is an example of the transcriptional complexity of chromosomal translocations between chr17 (BCAS3, RPS6KB1 and VMP1 in 17q23 and ABCA5 in 17q24) and chr20 (BCAS4, SULF2 and AIB1 in 20q13). These fusion products are likely due to the splicing process during transcription at this translocation region between chr17 and chr20. The fusion splices and fusion isoforms identified by IDP-fusion provide more evidence and data for further studies of transcription of this complicated genome rearrangement.

## DISCUSSION

Herein we report the first hybrid sequencing method, IDP-fusion, to characterize gene fusion events from RNA sequencing data at three levels: fusion gene detection, precise fusion site determination, and fusion isoform identification and quantification. In particular, we found multiple fusion sites from single fusion genes, many of which are likely caused by the alternative splicing during transcription and thus also referred to as fusion splices. The diversity of fusion splices and the regular splices of original genes can both drive the diversity of fusion isoform expression. The lack of a clear understanding of the diversity of isoform expression of fusion genes precludes investigation of the functions of fusion products, such as changes of abundance, protein coding sequence and chimeric domain combinations. Thus, IDP-fusion provides a comprehensive analysis to study gene fusion events.

The advantage of IDP-fusion is derived from the integration of the complementary strengths of TGS and SGS data. We have shown that the results achieved through hybrid sequencing are more accurate than either TGS-only or SGS-only methods (Figure 3A and Table 1). The unique alignments of TGS long reads are the basis of reliable fusion gene detection, which is not attained by increasing SGS coverage. In fact, the accuracy of fusion gene detection by IDP-fusion has no strong dependence on short reads. The F-score did not change significantly when changing the required number of supporting short reads (Supplementary Figure S7). In addition, long read alignments can uncover some fusion genes and fusion sites located in repetitive regions, which is challenging using SGS-only methods. For example, an unreported fusion site of the ENSG00000224738–VMP1 that is located in Short Interspersed Elements (SINE) was exclusively determined by IDP-fusion because single long reads can cover both SINE and some unique regions. TGS long reads can also detect fusion genes that contain novel genes and novel exons, such as AIB1–chr1:107078407 and UNK–ABCA5. The exon–intron structures of the unannotated regions can be clearly interpreted by long read alignment. Moreover, fusion genes are likely larger and contain more exons/splices than original genes, so the corresponding isoform identification is more complicated and difficult. TGS long reads can greatly improve isoform identification for long genes, which was shown in our previous work (22). However, precise fusion site determination requires SGS data. In addition, the large size of SGS data allows statistical modeling to estimate abundance of fusion isoforms. SGS data can also be used in error correction of TGS data to improve their mappability (20). Thus, the integration of SGS and TGS data through hybrid sequencing provides superior analytical ability as compared to either method alone.

## AVAILABILITY

IDP-fusion is released under the Apache 2.0 license. The source code and a reference manual are available at: http://www.healthcare.uiowa.edu/labs/au/IDP-fusion/.

## ACCESSION NUMBERS

The data is available on the National Center of Biotechnology Information database under the study accession number: PRJNA277461.

## SUPPLEMENTARY DATA

Supplementary Data are available at NAR Online.

SUPPLEMENTARY DATA
