# Supplementary material for: Characterization of fusion genes and the significantly expressed fusion isoforms in breast cancer by hybrid sequencing
Source: Nucleic Acids Res. 2015 Oct 10;43(18):e116. doi: 10.1093/nar/gkv562 (PMC4605286; doi:10.1093/nar/gkv562)
Supplement: SUPPLEMENTARY DATA [file supp_43_18_e116__index.html]

Characterization of fusion genes and the significantly expressed fusion isoforms in breast cancer by hybrid sequencing — Characterization of fusion genes and the significantly expressed fusion isoforms in breast cancer by hybrid sequencing — SUPPLEMENTARY DATA 

# Characterization of fusion genes and the significantly expressed fusion isoforms in breast cancer by hybrid sequencing

## SUPPLEMENTARY DATA

- SUPPLEMENTARY DATA
- SUPPLEMENTARY DATA
- SUPPLEMENTARY DATA
