# Supplementary material for: Characterization of fusion genes and the significantly expressed fusion isoforms in breast cancer by hybrid sequencing
Source: Nucleic Acids Res. 2015 Oct 10;43(18):e116. doi: 10.1093/nar/gkv562 (PMC4605286; doi:10.1093/nar/gkv562)

Supplementary Figure S1. *Structure of IDP-fusion*. IDP-fusion is executed through (1) a long read alignment and fusion candidate filtering process, (2) followed by the construction of an artificial reference sequence to be used for precise determination of fusion sites by short read alignment. (3) Long reads and short reads are used in detecting known isoforms, while (4) fusion splice linkage can be used to construct fusion isoform candidates. Both fusion and non-fusion isoforms undergo quantification and selection to produce a complete isoform output.

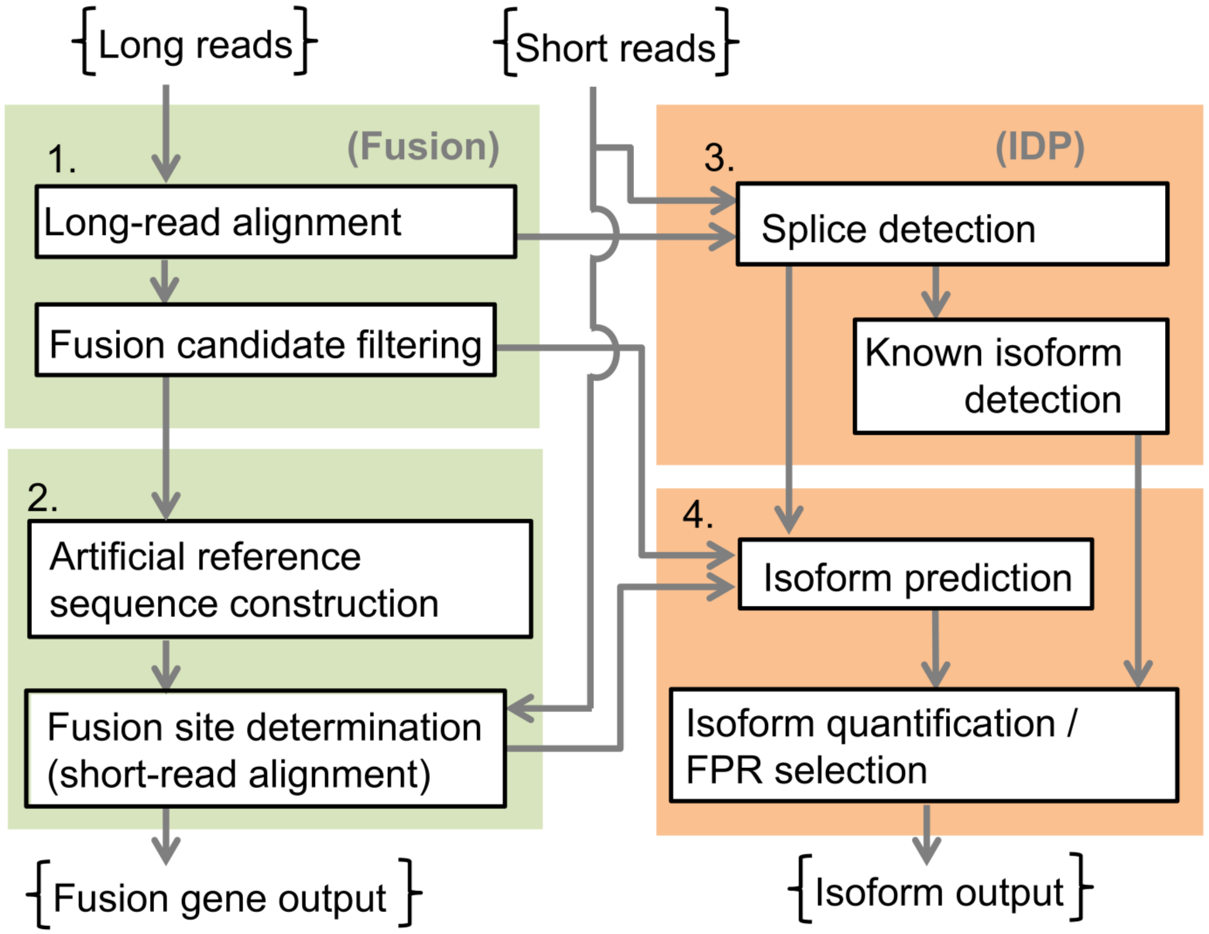

Supplementary Figure S2. *Overlap and gap of long read alignments*. Long reads could align in (1) continuous uniquely mapped segments, (2) however in some circumstances, a portion of a long read could share some overlap with another fragment of itself. (3) Still other times, a gap can exist between two uniquely mapped segments of a fusion. IDP-fusion can tolerate some gap or overlap in long read alignments when constructing fusion candidates (100bp tolerance by default).

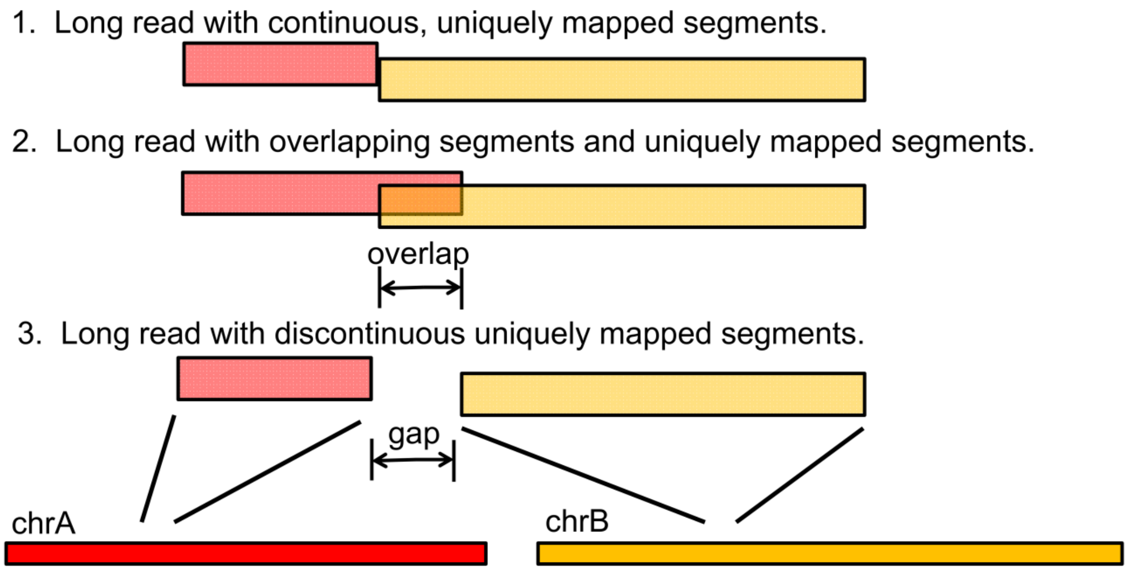

Supplementary Figure S3. *Artificial reference sequence (ARS) construction*. Pairs of uniquely mapped segments of long reads are made into artificial reference sequences (ARSs) to facilitate the determination of precise fusion sites via short read alignment. The flanking bases are added from the aligned regions and concatenating the sequences. Redundant ARSs are combined.

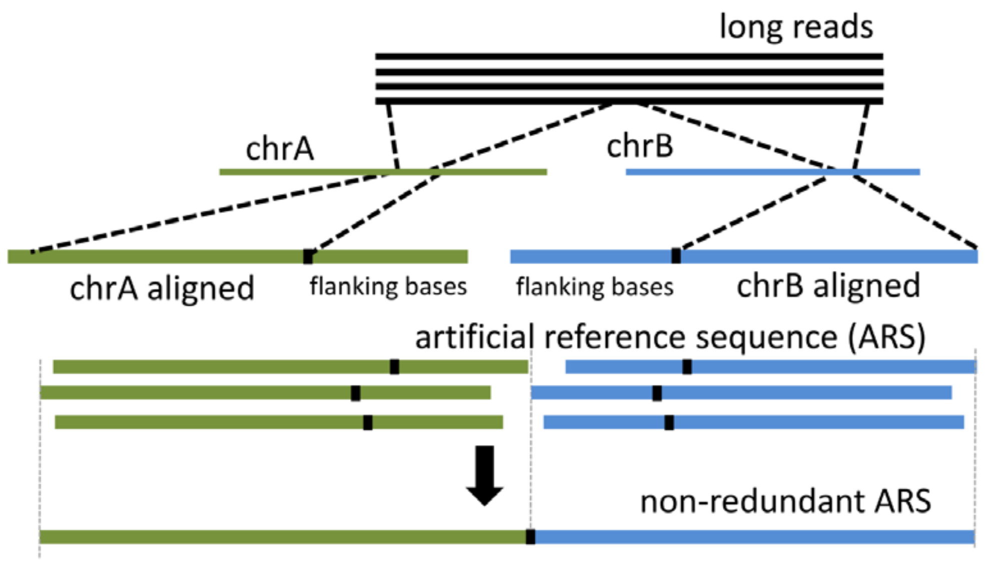

Supplementary Figure S4. *Non-redundant splice linkage.* (A) Multiple large gaps (>68bp) in a long read alignment are defined as splice linkage. (B) The exon boundary set of long read 2 $(p_6^-, p_7^+, p_8^-, p_8^+, p_9^-, p_{10}^+)$ is a subset of the one of long read 1 $(p_3^-, p_4^+, p_5^-, p_5^+, p_6^-, p_7^+, p_8^-, p_8^+, p_9^-, p_{10}^+)$, so long read 2 is redundant.

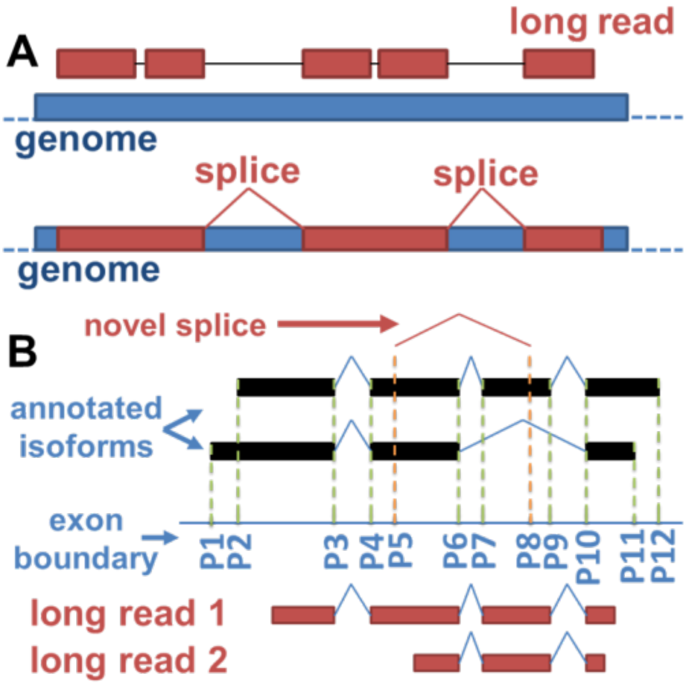

Supplementary Figure S5. *Two-Consecutive-Splice-Linkage definition of a true negative*. This illustration shows how an isoform with three or more exons can be decomposed into sets of Two-Consecutive-Splice-Linkages. When these are not present in any annotated database, the isoform they have come from is likely a true negative.

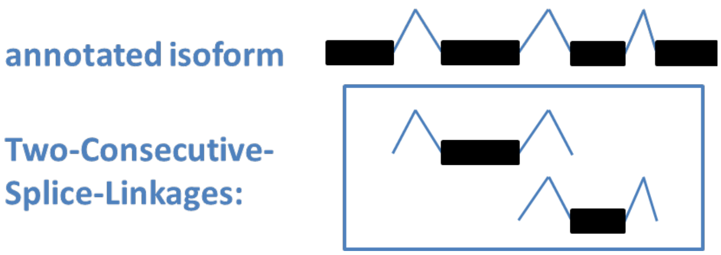

Supplementary Figure S6. *Numbers of false positive fusion sites detected when varying long and short read requirements*. The number of fusion sites detected by IDP-fusion in either MCF-7 or negative controls: A human embryonic stem cell line (hESC), brain, liver, and heart tissues. The number of detected fusion sites is shown when requiring at least one (A), two (B), or three (C) short reads, and varying the number of required supporting long reads from 1 to 10. While the largest drop in reported fusion sites among negative controls is seen when requiring at least two long reads rather than one, requiring at least two short reads rather than one also reduces false positives.

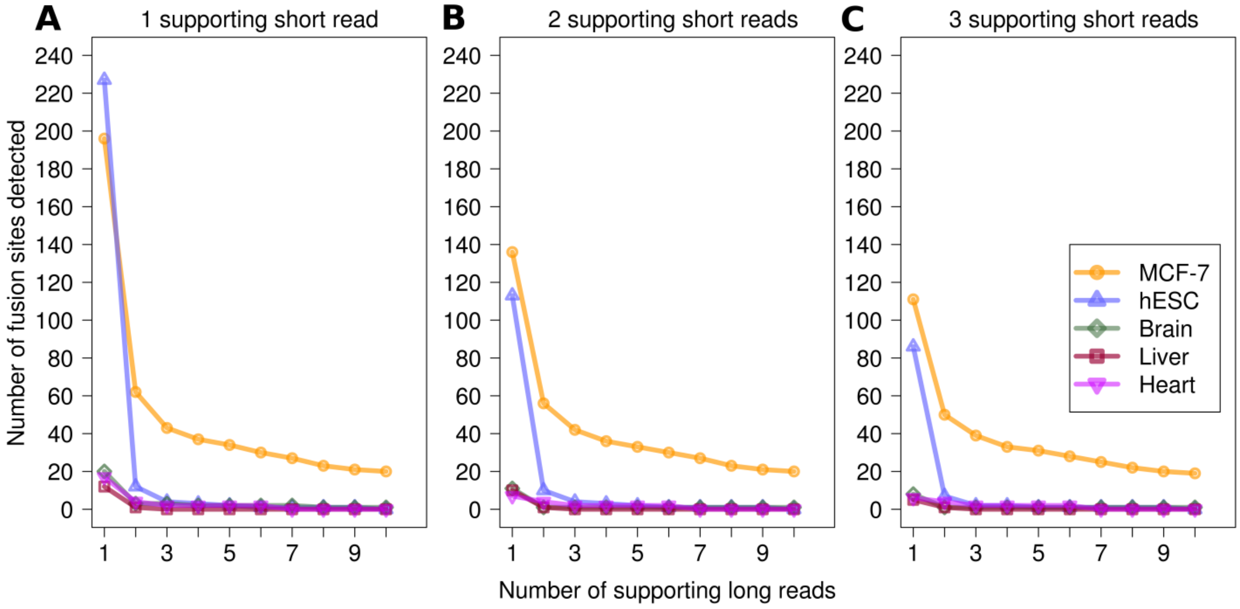

Supplementary Figure S7. *Accuracy (F-score) of fusion gene detection for different short read requirements.* The accuracy of fusion gene detection, as measured by F-score is shown for various minimum requirements of short read support of fusion sites. The maximal F-score of 0.4528 occurs when applying a requirement of at least 2 long reads and at least 2 short reads to be supporting fusion sites.

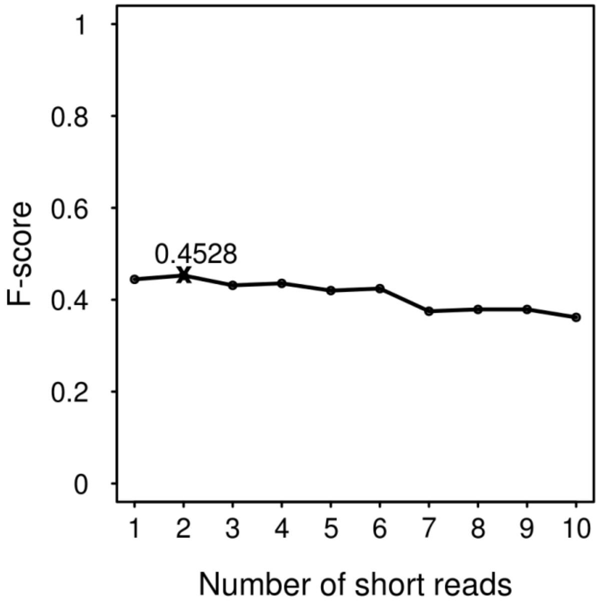

Supplement: SUPPLEMENTARY DATA [file supp_gkv562_nar-00852-met-k-2015-File010.pdf]
