# Supplementary material for: Characterization of fusion genes and the significantly expressed fusion isoforms in breast cancer by hybrid sequencing
Source: Nucleic Acids Res. 2015 Oct 10;43(18):e116. doi: 10.1093/nar/gkv562 (PMC4605286; doi:10.1093/nar/gkv562)
Supplement: SUPPLEMENTARY DATA [file supp_gkv562_nar-00852-met-k-2015-File011.pdf]

Characterization of fusion genes and the significantly expressed fusion isoforms in breast cancer by hybrid sequencing

Jason L Weirather, Pegah Tootoonchi Afshar, Tyson A Clark, Elizabeth Tseng, Linda S Powers, Jason Underwood, Joseph Zabner, Jonas Korlach, Wing Hung Wong, Kin Fai Au

SUPPLEMETARY MATERIALS AND METHODS

PacBio long read sequencing from breast cancer MCF-7 cells, human tissues and hESCs

RNA samples were purchased from Biochain Inc. (Newark, CA). For MCF-7, 200-500 ng of total RNA was used for reverse transcription. For the human tissue samples, approximately 500 ng of polyA selected RNA was used. Full-length, first strand cDNA was generated using the Clontech PCR cDNA Synthesis Kit. The cDNA was amplified with either Phusion (for MCF-7) or Kapa HiFi (for human tissues) polymerase followed by AMPure XP bead clean up.

Amplified cDNA was size fractionated by cutting bands from an agarose gel followed by DNA extraction with the QIAquick Gel Extraction Kit or by running the samples on a BluePippin. For MCF-7, three size bins were collected (1-2, 2-3, and 3-6 kb). Four size bins were collected for the human tissue samples (1-2, 2-3, 3-6, and 5-10 kb). Sequencing libraries were also generated from non-size selected cDNA from all samples. A second round of PCR amplification was carried out on each size fraction separately using either Phusion (for MCF-7) or Kapa HiFi (for human tissues) polymerase. Amplified PCR products were purified with AMPure XP beads.

SMRTbell libraries were generated from each amplified size fraction separately (1) using the SMRTbell Template Preparation Reagent Kit per the manufacturers' recommendations. 3-6 and 5-10 kb SMRTbell libraries were subjected to a second BluePippin size selection. Size selected SMRTbell templates were purified with AMPure XP beads. SMRTbell libraries were sequenced on a PacBio *RS* II using standard protocols. MCF-7 libraries were sequenced with P4-C2 chemistry using 90min or 2 hour movies. The human tissue SMRTbell libraries were sequenced with P4-C2 or P5-C3 chemistry using either 2 hour (1-2 and 2-3 kb) or 3 hour (3-6 and 5-10 kb) movies. The hybrid sequencing data of hESCs were downloaded from GSE51861 (2).

PacBio data pre-process and error correction by LSC

The CCS reads and the subreads were retrieved using the scripts of Pacific Biosciences SMRT-Analysis Software (v2.2.0). The CCS reads were constructed by the Consensus Tools script based on the PacBio P4-C2 chemistry with at least 2 full sequencing passes and a minimum predicted accuracy of 90%. Next, a set of higher quality set of CCS reads was generated with a minimum predicted accuracy of 95%. The subreads were extracted using the RS_Subreads.1 (v2.2.0) protocol, requiring a minimum sequence length of 50bp and a minimum read score of 75.

No error correction was performed for the CCS reads with ≥95% predicted accuracy. The CCS reads with 90-95% predicted accuracy were corrected by LSC (v1_beta). For molecules with no CCS reads generated, the longest subread was corrected by LSC.

LSC outputs the corrected-only region of the read as well as the full-length corrected read that includes corrected and uncorrected regions. To accurately count the number of independent reads supporting fusion sites, a non-redundant set of reads (one read per molecule) was created. If the corrected-only read was ≥90% the length of the full-length corrected read, the full-length corrected read was used in order to preserve polyadenylation sequences for transcript 3' end detection; otherwise the corrected-only sequence was used. The non-redundant set is comprised of 7,425,797 long reads made up of high-quality CCS reads and error-corrected reads by LSC. For the isoform identification and quantification, redundancy of the long reads is tolerated, because the quantitative model is only based on short read coverage. Therefore, when the length of corrected-only read was ≤90% of the full-length reads, both full-length corrected sequences and the corrected-only sequences are included. A total of 10,417,855 long reads were used to identify isoforms.

Validation by PCR, Sanger Sequencing and qPCR

PCR primers flanking the fusion site with an approximate melting temperature of 60 degrees were designed to produce an amplicon of 150 bp or less. The PCR reactions were performed with 250 pg of template from MCF-7 cDNA, as well as healthy breast cDNA, and genomic DNA as negative controls. AccuPrime Pfx Supermix (Invitrogen) in a 25ul volume was used to amplify the PCR product on a GeneAmp PCR System (ABI), with a denature at 95°C for 5 minutes followed by 95°C for 15 seconds, 55°C for 30 seconds and 68° C for 30 seconds for 35 cycles followed by 68°C extension of 7 minutes. The products were observed on a 2% agarose TAE gel. A second PCR reaction was performed using a 1:1000 dilution of that PCR product and run out on a 2% gel. Appropriately sized bands were then excised and purified using a Qiagen gel purification kit. Sanger sequencing was completed by the Iowa Institute of Human Genetics using the previously mentioned forward and reverse primers.

For BCAS4-BCAS3, the relative abundance of fusion splices was assessed with qPCR. Quantitative PCR was carried out using Fast SYBR Green Master Mix (ABI). Reactions were carried out in a 10 ul reaction volume with a 500 nanomolar primer concentration for 40 cycles on an ABI 7900 thermocycler at 95°C for 20 seconds to denature followed by 40 cycles of 95°C for 1 second and an annealing/extension phase at 60°C for 20 seconds. The relative expression of the fusion splice was determined by comparing the cycle threshold (Ct) of the product generated from 50 pg of MCF-7 cDNA template. To verify comparable efficiency of the qPCR reaction between different primers, standard curves were generated over 1:10, and 1:100 dilutions of the MCF-7 cDNA template.

Gene fusion detection by BreakFusion, deFuse, FusionMap, SOAPfuse, TopHat-Fusion TRUP, and Iso-Seq

All short read based gene fusion detection methods were run using the same paired-end MCF-7 short-read dataset. BreakFusion v1.0.1 (3) was run using BWA-mem v0.7.12 (4) under default settings, BreakDancer v1.1.2 (5) with a minimum mapping quality of 10, and BLAT v35 (6). The deFuse v0.6.2 software (7) was run using the configuration file included in the source and the outputs were the filtered results by the default setting. The FusionMap 2014-01-01 build (8) was run using default parameters in the control file, with no blacklist filter, reporting junctions with at least 4 “unique cutting positions”. SOAPfuse v1.26 (9) was run under its default settings utilizing a database constructed from the hg19 genome and Ensemble GRCh37.75 transcripts (10). TopHat-Fusion v0.1.0 (11) was run in two stages: 1) TopHat v2.0.11 (12) was run on the paired FASTQ files with the fusion-search option enabled and a minimum fusion distance of 100,000, and then 2.) TopHat-Fusion-post was run on the output using the default reporting of fusions with at least 3 supporting split reads, and 2 supporting mate pairs. TRUP (update 2015-03-19) (13) was run using SAMtools v1.2 (14), Bowtie2 v2.2.5 (15), BLAT v36 (6), GSNAP 2013-09-30 build (16), Velvet v1.2.10 (17), Oases v0.2.09 (18) and hg19 annotation database available on manual page. TRUP was run under default settings, except the maxIntron was set to 100,000 for fusion detection. The Iso-Seq pipeline from PacBio required a minimum of 5 full-length supporting long reads to identify a fusion.

REFERENCES

1. Travers, K.J., Chin, C.S., Rank, D.R., Eid, J.S. and Turner, S.W. (2010) A flexible and efficient template format for circular consensus sequencing and SNP detection. *Nucleic acids research*, **38**, e159.
2. Au, K.F., Sebastiano, V., Afshar, P.T., Durruthy, J.D., Lee, L., Williams, B.A., van Bakel, H., Schadt, E.E., Reijo-Pera, R.A., Underwood, J.G. *et al.* (2013) Characterization of the human ESC transcriptome by hybrid sequencing. *Proceedings of the National Academy of Sciences of the United States of America*, **110**, E4821-4830.
3. Chen, K., Wallis, J.W., Kandoth, C., Kalicki-Veizer, J.M., Mungall, K.L., Mungall, A.J., Jones, S.J., Marra, M.A., Ley, T.J., Mardis, E.R. *et al.* (2012) BreakFusion: targeted assembly-based identification of gene fusions in whole transcriptome paired-end sequencing data. *Bioinformatics*, **28**, 1923-1924.
4. Li, H. and Durbin, R. (2009) Fast and accurate short read alignment with Burrows-Wheeler transform. *Bioinformatics*, **25**, 1754-1760.
5. Fan, X., Abbott, T.E., Larson, D. and Chen, K. (2014) BreakDancer - Identification of Genomic Structural Variation from Paired-End Read Mapping. *Curr Protoc Bioinformatics*, **2014**.
6. Kent, W.J. (2002) BLAT--the BLAST-like alignment tool. *Genome research*, **12**, 656-664.
7. McPherson, A., Hormozdiari, F., Zayed, A., Giuliany, R., Ha, G., Sun, M.G., Griffith, M., Heravi Moussavi, A., Senz, J., Melnyk, N. *et al.* (2011) deFuse: an algorithm for gene fusion discovery in tumor RNA-Seq data. *PLoS computational biology*, **7**, e1001138.
8. Ge, H., Liu, K., Juan, T., Fang, F., Newman, M. and Hoeck, W. (2011) FusionMap: detecting fusion genes from next-generation sequencing data at base-pair resolution. *Bioinformatics*, **27**, 1922-1928.

9. Jia, W., Qiu, K., He, M., Song, P., Zhou, Q., Zhou, F., Yu, Y., Zhu, D., Nickerson, M.L., Wan, S. *et al.* (2013) SOAPfuse: an algorithm for identifying fusion transcripts from paired-end RNA-Seq data. *Genome biology*, **14**, R12.
10. Flicek, P., Amode, M.R., Barrell, D., Beal, K., Brent, S., Carvalho-Silva, D., Clapham, P., Coates, G., Fairley, S., Fitzgerald, S. *et al.* (2012) Ensembl 2012. *Nucleic acids research*, **40**, D84-90.
11. Kim, D. and Salzberg, S.L. (2011) TopHat-Fusion: an algorithm for discovery of novel fusion transcripts. *Genome biology*, **12**, R72.
12. Trapnell, C., Pachter, L. and Salzberg, S.L. (2009) TopHat: discovering splice junctions with RNA-Seq. *Bioinformatics*, **25**, 1105-1111.
13. Fernandez-Cuesta, L., Sun, R., Menon, R., George, J., Lorenz, S., Meza-Zepeda, L.A., Peifer, M., Plenker, D., Heuckmann, J.M., Leenders, F. *et al.* (2015) Identification of novel fusion genes in lung cancer using breakpoint assembly of transcriptome sequencing data. *Genome biology*, **16**, 7.
14. Li, H., Handsaker, B., Wysoker, A., Fennell, T., Ruan, J., Homer, N., Marth, G., Abecasis, G., Durbin, R. and Genome Project Data Processing, S. (2009) The Sequence Alignment/Map format and SAMtools. *Bioinformatics*, **25**, 2078-2079.
15. Langmead, B. and Salzberg, S.L. (2012) Fast gapped-read alignment with Bowtie 2. *Nature methods*, **9**, 357-359.
16. Wu, T.D. and Nacu, S. (2010) Fast and SNP-tolerant detection of complex variants and splicing in short reads. *Bioinformatics*, **26**, 873-881.
17. Zerbino, D.R. and Birney, E. (2008) Velvet: algorithms for de novo short read assembly using de Bruijn graphs. *Genome research*, **18**, 821-829.
18. Schulz, M.H., Zerbino, D.R., Vingron, M. and Birney, E. (2012) Oases: robust de novo RNA-seq assembly across the dynamic range of expression levels. *Bioinformatics*, **28**, 1086-1092.
